# Buffalo Medical and Surgical Association

**Published:** 1883-08

**Authors:** Clayton M. Daniels

**Affiliations:** Secretary


### Buffalo Medical and Surgical Association.

*Regular Meeting, July 3d, 1883.*

Vice-President, Dr. F. W. Bartlett, in the Chair. Dr. C. M. Daniels, Secretary.

Present—Drs. Frank, Weil, Gay, Hartwig, Coakley, Hopkins, Grove and Fell.

*Voluntary Communications*—Being in order, Dr. Frank reported a case of epilepsy of several years' standing, the paroxysms occurring from one to three times weekly; patient had also spermatorrhœa with phymosis. Operation for phymosis resulted in the immediate cessation of epilepsy, and four months had now passed with no recurrence of the symptoms.

Dr. Gay, in commenting upon the case, said it was very interesting and important, and that some further experiments in this direction were desirable. Also mentioned a case of a child of eight years having epilepsy to a degree approaching idiocy, who was constantly irritating penis. The hands were secured so as to prevent it, and several months after there was marked improvement in the condition.

Dr. Hartwig stated that although it might be circumstantial only, that there was no epilepsy among the Jews, so far as he was able to ascertain. The fact seemed to give more importance to the case reported by Dr. Frank.

Dr. Daniels spoke of a case where a boy was injured by being thrown from a train, the right temporal bone being perforated, with oozing of brain substance, producing complete insensibility, the only movement being the involuntary act of frequently grasping the penis. Death occured twenty-four hours after injury.

Dr. Grove reported a case, and showed a shawl pin of brass, two inches in length, bearing a head one-quarter inch in diameter, which was accidentally swallowed by Miss L. After the course of four and one-half days, during which time considerable pain in stomach and œsophagus was experienced, in a siege of coughing, the foreign body was brought to light. The point of this pin was exceedingly sharp, and was supposed, from symptoms of the patient, to have penetrated, partially, at least, the walls of the œsophagus. The question of interest was, should emetics in such a case be administered?

Dr. Gay reported a case of apoplexy; convulsions severe; patient pulseless; insensible and had paralysis to a great degree. He bled freely from arm, which immediately stopped the convulsions and restored sensibility. Although only four days had elapsed, the patient was gradually improving, with good prospects for recovery. He wished to call the attention of the profession to the necessity for the use of the lancet, believing it should be used more frequently.

Dr. Bartlett thought we all said that blood letting was coming in vogue. He had bled quite a number, and with benefit. Related a case of delirium tremens, where a man came to his office and insisted on being bled, which being done resulted in an immediate improvement.

Dr. Hartwig gave an interesting account of an ovarian tumor which he recently removed, being a multiple cystoid weighing something over sixty pounds; patient recovered. The history of the case and complications given in detail would be too long for this report, and would form an interesting subject for a separate article.

Dr. Gay referred to carbolic acid, as spoken of by Dr. Hartwig, and believed it dangerous when used in strong solution, and that such was now the general opinion of the profession.

Dr. Gay reported a case of injury to the tarsus, being crushed by car wheel; attempt to save foot was unsuccessful; amputation of leg, and patient placed in tent outside of hospital; Esmarch's bandages used, and boroglyceride dressing; obtained union by first intention; recommended the outdoor treatment as being very beneficial; several other cases having similar conditions that were being treated in the tents at the hospital were all doing equally well.

Dr. Bartlett reported a case of "fœtal malformation." It will appear in detail in the September number of the JOURNAL.

Dr. Fell, as representative of the "American Society of Microscopists," cordially invited the members of this association to attend their annual meeting at Chicago, beginning August 7th.

*Prevailing Diseases.*—None reported, the city being in an unusually healthful condition.

Adjourned. CLAYTON M. DANIELS, *Secretary.*

